# Supplementary material for: Chromothripsis during telomere crisis is independent of NHEJ, and consistent with a replicative origin
Source: Genome Res. 2019 May;29(5):737–49. doi: 10.1101/gr.240705.118 (PMC6499312; doi:10.1101/gr.240705.118)
Supplement: Supplemental Material [file supp_gr.240705.118_Supplemental_file_1.zip › contigs/annotated_contigs/DB73/contig.2.DB73_length_597_mean_cov_8.18425460637.docx]

**DB73_length_597_mean_cov_8.18425460637**

```
G|CAGGTGTACAAGGCTATCAAATTTATTGAATATCGTTTAGGTAGATCATTCAGTTCCATTACTCTATTAATACTGAAATTCTGTAAC
  >chr3:82280132-82280641 + E=6e-292 p=1e-02
TGTACTGTGAGACATTTGTCTAACATTGACTTCTTATTGGCAGATTCAATACCTGTTGACTTACGATAAATTTTAAGGTTTGCAGATTG

TTGAATATGTCTGAAAGTTCTTTGAGAAGAAGAATATTAGGAAACAGCTGAAGTGCTTTTAAACTATGATACAGTCTTTTGGCATGCTA

ATGAATTGAACTAGAAAACTCGTTTGCATCTTAAAGCCACAAATCATAAACTGTGAATGAAAAACCAACCATGAAGGAAAAAAAATTGG

TGTGTTCTGTTATAAGTTAGTATGAAAGTCTACATTTTGTCTAGTGATAAACAACACTCACTGCAGATACTTCAGGTTAGACCAGATCT

GGTCTGAAAGCTTAGCAGATTGCACTCTAATGAGGGATTCTGGGGAGATGATTAGCTATAAGGACA|ACATCAAG|CCACTTGGTCCAG
                                                                           >chr3:802978
AGCTGAGTTCAAGTCCTGAATATCTTTGTTAATTTTCTGTCTCAATGATCTGTCTAACAT|CCTGAA45-80297918 - E=3e-32
```
